# Distal radial artery cannulation in the anatomical snuffbox is useful for arterial blood pressure monitoring in neurosurgery: a case report

**DOI:** 10.1186/s40981-020-00365-0

**Published:** 2020-08-01

**Authors:** Ryusuke Tanaka, Tohru Shiratori, Chiaki Kiuchi, Junichi Sasao

**Affiliations:** Division of Anesthesiology, Ina Central Hospital, 1313-1 Koshiroukubo, Ina City, Nagano, 396-8555 Japan

To the Editor:

Conventional radial artery (RA) cannulation at the wrist interferes with electrode placement for intraoperative neurophysiological monitoring (IONM) and is often troubled by dampened arterial waveforms after postural changes. We report the use of distal radial artery (DRA) cannulation in the anatomical snuffbox for arterial blood pressure (ABP) monitoring during a spine surgery requiring IONM and postural changes.

An 89-year-old man underwent extreme lateral interbody fusion in the right lateral decubitus and prone positions with motor evoked potential (MEP) monitoring. After inducing anesthesia with propofol and remifentanil, his wrist was adducted on a roll with the point of cannulation facing upwards. We successfully inserted a 22-gauge cannula (Surflo; TERUMO, Tokyo, Japan) in the DRA in the anatomical snuffbox on the first attempt under ultrasound guidance (Fig. 1a). This technique of DRA cannulation provided good ABP monitoring throughout surgery.

**Fig. 1:**
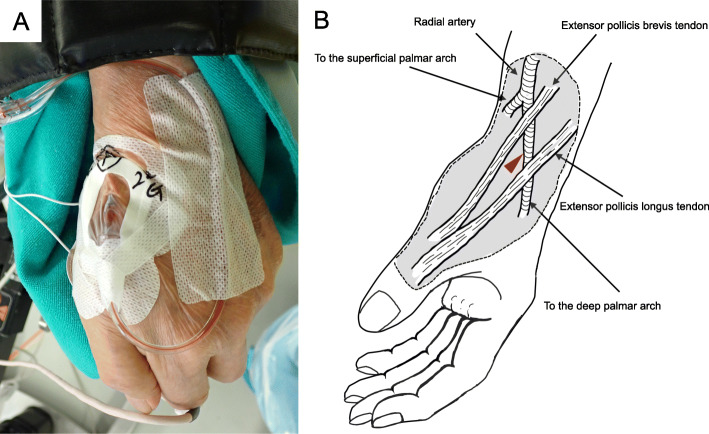
**a** Distal radial artery cannulation in the anatomical snuffbox. An electrode for recording the motor evoked potential from the abductor pollicis brevis is attached to the thenar area. The left forearm is pronated and fixed on a padded armrest in the right lateral decubitus position. **b** Anatomical scheme of the entry point for cannulation of the dorsal radial artery of the left hand. The entry point is indicated by the red arrowhead

In recent times, the DRA has been increasingly cannulated for cardiac and neurosurgical interventions [1, 2]. Based on our experience, the advantages of using the DRA for ABP monitoring in neurosurgery are as follows: (1) the stimulating electrodes for the assessment of somatosensory evoked potentials and the recording electrodes for MEP are placed above the median nerve at the wrist and the abductor pollicis brevis, respectively. Thus, RA cannulation at the wrist interferes with the proper placement of these electrodes. DRA cannulation avoids this interference and enables the adequate stimulation and recording that is necessary for reliable IONM. (2) The arterial waveform is stable in the prone and lateral decubitus positions because DRA cannulation in the anatomical snuffbox permits pronation of the patient’s forearm. In the prone and lateral decubitus positions, the palmar aspects of the pronated arms face the padded armrests. This frequently dampens the arterial waveform monitored by the conventional RA cannulation method. Additionally, the arterial wave forms obtained by cannulation of the DRA are less affected by wrist flexion during postural changes than those obtained by conventional RA cannulation [3].

The DRA is distal to the division of the superficial palmar branch of the RA (Fig. 1b). It continues as the deep palmar arch and receives collateral flow from the superficial palmar branch and the ulnar artery [2, 4]. Therefore, DRA cannulation confers lesser risk of ischemia compared to the conventional technique of RA cannulation [2, 4]. It has also been reported that the occurrence of other complications, including hematomas and neuropathy, did not significantly differ between these two approaches [1, 3]. However, the possibility of nerve injury should be considered because the superficial branch of the radial nerve is near the entry point of DRA cannulation [3]. Further well-designed studies are needed for ensuring the safety of DRA cannulation during the perioperative period. In addition, the DRA may be more difficult to cannulate than the RA due to its smaller diameter [1], which is a limitation of DRA cannulation.

In conclusion, the DRA in the anatomical snuffbox can be a useful site for ABP monitoring in neurosurgery and enables adequate electrode positioning for neurophysiological assessments.

## Data Availability

Not applicable

## Abbreviations

ABP: Arterial blood pressure
DRA: Distal radial artery
IONM: Intraoperative neurophysiological monitoring
MEP: Motor evoked potential
RA: Radial artery
